# Human G protein-coupled receptor 30 is *N*-glycosylated and N-terminal domain asparagine 44 is required for receptor structure and activity

**DOI:** 10.1042/BSR20182436

**Published:** 2019-02-26

**Authors:** Ernesto Gonzalez de Valdivia, Caroline Sandén, Robin Kahn, Björn Olde, L.M. Fredrik Leeb-Lundberg

**Affiliations:** 1 Department of Experimental Medical Science, Lund University, Lund 22184, Sweden; 2 Department of Pediatrics, Lund University, Lund 22184, Sweden; 3 Wallenberg Center for Molecular Medicine, Lund University, Lund 22184, Sweden; 4 Department of Cardiology, Lund University, Lund 22184, Sweden

**Keywords:** extracellular signal-regulated kinases, estrogens, endocytosis, G-protein-coupled receptors, glycosylation, trafficking

## Abstract

G protein-coupled receptor 30 (GPR30), or G protein-coupled estrogen receptor (GPER), is a G protein-coupled receptor (GPCR) that is currently attracting considerable attention in breast cancer and cardiometabolic regulation. The receptor was reported to be a novel membrane estrogen receptor mediating rapid non-genomic responses. However, questions remain about both the cognate ligand and the subcellular localization of receptor activity. Here, we used human embryonic kidney (HEK) 293 (HEK293) cells ectopically expressing N-terminally FLAG-tagged human GPR30 and three unique antibodies (Ab) specifically targetting the receptor N-terminal domain (N-domain) to investigate the role of *N*-glycosylation in receptor maturation and activity, the latter assayed by constitutive receptor-stimulated extracellular-regulated protein kinase (ERK) 1/2 (ERK1/2) activity. GPR30 expression was complex with receptor species spanning from approximately 40 kDa to higher molecular masses and localized in the endoplasmatic reticulum (ER), the plasma membrane (PM), and endocytic vesicles. The receptor contains three conserved asparagines, Asn^25^, Asn^32^, and Asn^44^, in consensus *N*-glycosylation motifs, all in the N-domain, and PNGase F treatment showed that at least one of them is *N*-glycosylated. Mutating Asn^44^ to isoleucine inactivated the receptor, yielding a unique receptor species at approximately 20 kDa that was recognized by Ab only in a denatured state. On the other hand, mutating Asn^25^ or Asn^32^ either individually or in combination, or truncating successively N-domain residues 1–42, had no significant effect either on receptor structure, maturation, or activity. Thus, Asn^44^ in the GPR30 N-domain is required for receptor structure and activity, whereas N-domain residues 1–42, including specifically Asn^25^ and Asn^32^, do not play any major structural or functional role(s).

## Introduction

The topology of G protein-coupled receptors (GPCR) is highly conserved, comprising an N-terminal extracellular domain (N-domain), seven transmembrane helices linked by three extracellular loops and three intracellular loops, and a C-terminal intracellular domain [1]. The N-domain is considered important for receptor maturation from the endoplasmatic reticulum (ER) to the plasma membrane (PM). Most mammalian GPCRs contain one or more asparagines in consensus motifs for *N*-linked glycosylation (Asn–X–Ser/Thr) in this domain, and glycosylation of these residues, the most common covalent receptor modification, is believed to assist in correctly orienting the receptor in the ER membrane. However, the precise role of *N*-glycosylation in receptor maturation is not clear, as mutating such asparagines interferes with PM expression of some GPCR but not others [2]. Beyond this role, the N-domain also serves specific functional roles in some GPCRs. In class B and C GPCRs the N-domain contributes directly to ligand binding, in class A protease-activated receptors it is proteolytically cleaved, exposing a tethered ligand that activates the receptor, and in adhesion GPCR it harbors autoproteolytic activity, generating a tethered ligand that antagonizes constitutive receptor activity [3].

GPCR 30 (GPR30) is a relatively recently identified class A GPCR, and growing evidence suggests that the receptor participates in breast cancer and cardiometabolic regulation. Originally believed to be a peptide receptor based on structural homology with chemokine receptors [4], GPR30 was subsequently reported to mediate rapid non-genomic estrogen response [5,6], thus renaming the receptor G protein-coupled estrogen receptor (GPER). However, several groups have failed to show estrogen-stimulated GPR30 activity in defined recombinant model systems, leading some investigators to question the identity of the cognate receptor ligand and instead monitor constitutive receptor activity [7,8]. Another outstanding issue concerns the subcellular localization of GPR30, which is complex with receptors reported both in the PM and in intracellular membranes in both native and recombinant cell systems [5–18], and receptor activity proposed to occur both in the PM [5,7,8,17], ER [6], and nucleus [18]. Obviously, critical details are missing regarding the GPR30 pharmacological profile, subcellular localization, and effector coupling.

GPR30 activity in the PM is best described, whereas very little to no details are available for activity in the ER or the nucleus. In the PM, the receptor couples primarily to Gi/o and increased extracellular-regulated protein kinase (ERK) 1/2 (ERK1/2) and phosphoinositide 3-kinase (PI3K) activity [5,8,19–21], but some evidence also indicate coupling to Gs and increased cAMP levels [5]. In addition, the receptor contains a postsynaptic density-95 (PSD-95)/Discs-large/ZO-1 homology (PDZ) motif at the C-terminus through which it couples to the membrane-associated guanylate kinase (MAGUK) scaffold proteins synapse-associated protein 97 (SAP97) and PSD-95 [7,8,16,17], and in turn to protein kinase A-anchoring protein 5 (AKAP5) [7,8]. The PDZ-dependent interaction retains the receptor in the PM [7,16], favors Gi/o-dependent stimulation of ERK1/2 activity [8], and mediates Gi/o-independent inhibition of cAMP production [7] and PM Ca^2+^-ATPase 4b activity [17]. Thus, MAGUK proteins appear to regulate both the subcellular localization and function of the receptor.

To further address the subcellular localization of GPR30 function, we investigated the role of the human GPR30 N-domain in human embryonic kidney (HEK) 293 (HEK293) cells ectopically expressing the receptor. We focussed primarily on the roles of three conserved consensus *N*-glycosylation sites in this domain, Asn^25^, Asn^32^, and Asn^44^. Using three specific N-domain antibodies (Ab) and constitutive stimulation of ERK1/2 activity to monitor the receptor, we show that Asn^44^ is critical for receptor structure and activity, whereas residues 1–42, including specifically Asn^25^ and Asn^32^, do not play any major structural or functional role(s).

## Materials and methods

### Cell culture and DNA constructs

HEK293 cells (American Type Culture Collection, Manassas, VA) were grown in Phenol Red-free Dulbecco’s modified Eagle’s medium (DMEM) supplemented with 10% FBS in 10% CO_2_ at 37°C. Human GPR30 cDNA was subcloned into the pcDNA3.1 vector. An N-terminal artificial signal sequence, as previously described [22,23], the FLAG sequence, and a short linker sequence were added in series. The GPR30 mutations N25I, N32I, N44I, and N25/32I, and truncations del23, del31, and del43 were introduced using Phusion polymerase-based site-directed mutagenesis essentially as previously described [24]. Transient plasmid transfection of cells was done using TransIT-LT1 transfection reagent (Mirus Bio LLC, Madison, WI), whereas stable plasmid transfection was done using the calcium phosphate precipitate method, and stable transfectants were selected using zeocin resistance.

### Immunoprecipitation and immunoblotting

**Figure 1:**
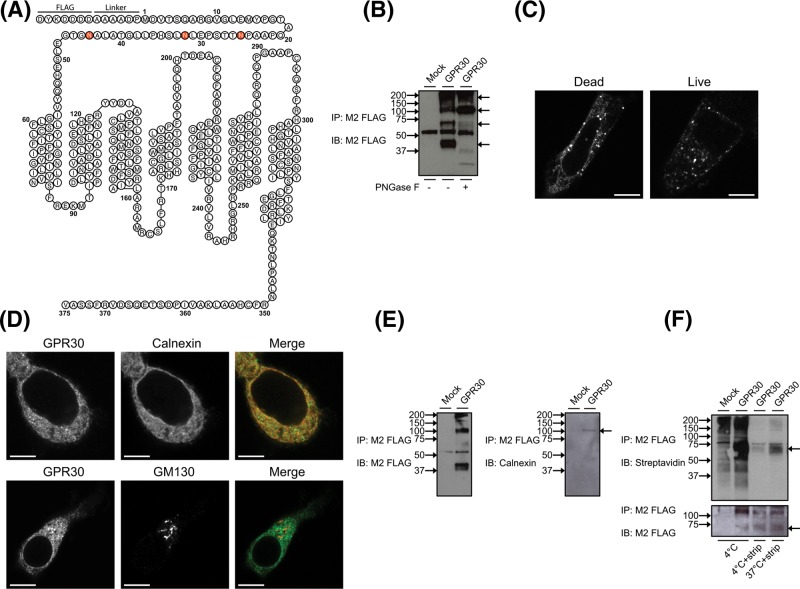
GPR30 *N*-glycosylation and subcellular distribution (**A**) Schematic depiction of the FLAG-tagged human GPR30 construct used in the present study. (**B**) HEK cells transfected without (*Mock*) and with GPR30 were immunoprecipitated (*IP*) with M2 FLAG Ab beads, treated without (−) and with (+) PNGase F, and then immunoblotted (*IB*) with M2 FLAG Ab. (**C**) HEK cells transfected with GPR30 were fixed and permeabilized prior to incubation with M1 FLAG Ab (*Dead*) or preincubated live with M1 FLAG Ab for 30 min at 37°C prior to fixation and permeabilization (*Live*). (**D**) HEK cells transfected with GPR30 were fixed and permeabilized prior to incubation with M1 FLAG Ab (*GPR30*) together with calnexin Ab or GM130 Ab. (**E**) HEK cells transfected without (*Mock*) and with GPR30 were IP with M2 FLAG Ab beads and then IB with M2 FLAG Ab or calnexin Ab. (**F**) HEK cells transfected without (*Mock*) and with GPR30 were labeled with disulphide-cleavable sulpho-NHS-SS-biotin at 4°C and then treated without or with glutathione (*strip*) at 4°C or preincubated at 37°C for 30 min prior to glutathione treatment. Cell lysates were then IP with M2 FLAG Ab beads followed by IB with M2 FLAG Ab or streptavidin-HRP. In (B,E,F), molecular mass standards (*left side arrows*) and specific protein species (*right side arrows*) as discussed in ‘Results’ section are indicated, and the results are representative of experiments performed at least three times. In (C,D), cells were subsequently incubated with secondary rabbit anti-mouse or mouse anti-rabbit ALEXA488- or ALEXA568-labeled Ab. The individual and merged (*Merge*) images were collected using a Nikon Eclipse confocal microscope, 60× objective. The results are representative of experiments performed at least three times. *Bar*, 10 μm.

Confluent cells were washed twice with ice-cold PBS and lysed in 0.2–1 ml lysis buffer (1% NP-40, 0.5% deoxycholate, 0.1% SDS, 50 mM Tris/HCl, pH 7.4, 150 mM NaCl, 5 mM EDTA, 10 mM NaF, 10 mM Na_2_HPO_4_) with complete protease inhibitor cocktail (Sigma–Aldrich, St. Louis, MO). Lysates were cleared by centrifugation at 10000×***g*** for 10 min at 4°C. Proteins were denatured in SDS/PAGE sample buffer including 6% β-mercaptoethanol for 30 min at 37°C. Equal amounts of protein were added to each lane of a polyacrylamide gel (10–20 μg protein), fractionated by SDS/PAGE, transferred to a nitrocellulose membrane, and the membrane was blocked for at least 30 min in TBS and 5% nonfat milk. Blots were stained with mouse M2 FLAG antibody (Ab) (Sigma–Aldrich; 1:1000), goat GPR30 Ab (R&D Systems, Minneapolis, MN; 1:200), or rabbit calnexin Ab (Sigma–Aldrich; 1:2000). Immunoreactive bands were visualized with a chemiluminescence immunodetection kit using peroxidase-labeled Ab according to the procedure described by the supplier (PerkinElmer Life and Analytical Sciences, Waltham, MA). Some blots were stripped by washing in 62.5 mM Tris/HCl, pH 6.7, 2% SDS, and 100 mM β-mercaptoethanol for 30 min at 50°C, washed three times for 10 min each in TBS, and then restained with Ab. Blots were then exposed to film and developed (Figure 1B,E,F), or scanned using a Chemidoc XRS+ imager (Bio-Rad, Hercules, CA) (Figure 3C–E). In some cases, GPR30 was immunoprecipitated prior to immunoblotting by incubating the cleared lysates with mouse M2 FLAG Ab affinity resin (Sigma–Aldrich) overnight at 4°C. The precipitates were washed extensively and sequentially in the lysis buffer and 10 mM Tris/HCl, pH 7.4 and then subjected to immunoblotting as described above.

### ERK activity

ERK1/2 activity was assayed by immunoblotting as described above using phospho-ERK1/2 (pERK) Ab (Santa Cruz Biotechnology, Santa Cruz, CA; 1:200) for ERK1/2 phosphorylation and ERK1/2 (ERK) Ab (Santa Cruz Biotechnology; 1:1000) for total ERK1/2. Briefly, cells were grown to confluence in 60-mm dishes in DMEM with 10% FBS, washed, incubated without serum for 1 h. The cells were then washed, lysed, and subjected to immunoblotting, and immunoreactive bands were visualized as described above. The combined band densities of ERK1 and ERK2 were quantitated using ImageJ software (version 1.48v), and ERK1/2 activity was expressed as the ratio between the combined pERK band densities and the combined ERK band densities for each condition.

### Enzymatic deglycosylation

To determine the presence of *N*-glycosylation in GPR30, receptor immunoprecipitates were treated with 500 units of PNGase F (New England Biolabs, Ipswich, MA) in 10 mM Tris/HCl, pH 7.4, for 2 h at 37°C.

### Cell surface biotinylation

Confluent cells were washed twice with ice-cold PBS and then incubated with 0.3 mg/ml disulphide-cleavable sulpho-NHS-SS-biotin (Thermo Fisher Scientific) in PBS for 30 min at 4°C with gentle agitation. The cells were then washed twice with TBS to quench the biotinylation reaction. Cells were subsequently extracted in lysis buffer containing 1 mg/ml iodoacetamide and complete protease inhibitor cocktail, and cell debris was removed by centrifugation at 10000×***g*** for 10 min at 4°C. GPR30 was then immunoprecipitated by incubating with mouse M2 FLAG Ab affinity resin. Proteins were denatured in SDS/PAGE sample buffer without reducing agent followed by SDS/PAGE as described above. Biotinylated proteins were visualized by incubating with the Vectastain avidin-biotinylated enzyme complex immunoperoxidase reagent (Vector Laboratories, Burlingame, CA) followed by development with the chemiluminescence immunodetection kit.

### Immunofluorescence microscopy

Cells were propagated to 50% confluency in growth medium on glass coverslips, coated with poly-d-lysine (Sigma–Aldrich) or 0.1% gelatin (Sigma–Aldrich), and then incubated in serum-free medium for at least 1 h before treatment. To monitor specifically cell surface and internalized GPR30 (*Live*), we took advantage of the fact that mouse M1 FLAG Ab (Sigma–Aldrich; 1:500) labels specifically the receptor extracellular N-terminal FLAG epitope. Therefore, ‘feeding’ live transfected cells with this Ab for 30 min at 37°C monitored exclusively cell surface receptor–Ab complexes and complexes that had subsequently undergone endocytosis. Cells were then fixed using 3.7% formaldehyde in PBS and permeabilized with blotto (3% dry milk, 0.1% Triton X-100, 1 mM CaCl_2_, 50 mM Tris/HCl pH 7.4). To monitor total cellular GPR30, cells were fixed (*Dead*), permeabilized, and then incubated in blotto with mouse M1 FLAG Ab (1:500) or goat GPR30 Ab (1:100) for 1 h at 22°C. In all experiments, cells were then washed with PBS and receptors visualized by incubation with secondary Alexa488-labeled anti-mouse IgG2b Ab or Alexa488-labeled anti-goat Ab (Life Technologies, Carlsbad, CA). For colocalization studies with GPR30, fixed and permeabilized cells were also incubated for 1 h at 22°C with either mouse H4A3 (LAMP1) Ab (Developmental Studies Hybridoma Data Bank; 1:250), mouse EEA1 Ab (BD Biosciences; 1:500), rabbit calnexin Ab (Sigma–Aldrich; 1:200), or mouse anti-GM130 Ab (BD Biosciences, San Jose, CA; 1:250). Alexa568-labeled anti-mouse or anti-rabbit Ab (Invitrogen, Carlsbad, CA) were then used as secondary Ab. For colocalization of internalized receptor with transferrin, live cells were labeled with mouse M1 FLAG Ab for 60 min at 37°C, and with the addition of Alexa568-labeled transferrin (Thermo Fisher Scientific; 1:500) during the last 30 min. In some experiments, DAPI was used for nuclear staining. Images were acquired using a Nikon C1 laser scanning confocal microscope mounted on a Nikon Eclipse TE2000-E, with pinhole size 30 μm and CFI60 Plan APO VC 60× 1.40 NA oil immersion objective. Twenty fields of view were employed for microscopic evaluation.

### Flow cytometry

Cells were fixed in 2% paraformaldehyde for 15 min and then washed with PBS with Ca^2+^/Mg^2+^. The cells were then incubated with primary mouse M1 FLAG Ab (1:200), goat GPR30 Ab (1:100), or mouse IgG (DAKO, Glostrup, Denmark) for 20 min with and without 0.2% Triton-X100/PBS (Santa Cruz Biotechnology) at room temperature, to detect intracellular and cell surface expression of receptors, respectively. Cells were then washed with PBS with Ca^2+^/Mg^2+^, resuspended in PBS with Ca^2+^/Mg^2+^ and incubated with phycoerythrin-labeled goat anti-mouse Ab (DAKO; 1:2000) or Alexa488-labeled donkey anti-goat Ab (Thermo Fisher Scientific; 1:1000) as secondary Ab for 20 min at room temperature in the dark, and then analyzed by flow cytometry. The specificity of the secondary Ab was tested by omitting the primary Ab. The cells were analyzed using a BD FACSCanto II Cytometer and FACSDiva Software (Beckton Dickinson Immunocytometry Systems, San Jose, CA). In all experiments, the background fluorescence was set according to the control Ab and percent positive cells were calculated.

### Data analysis

Data are presented as means ± S.E.M. Student’s two-tailed *t* test for unpaired data was done to evaluate statistical significance. *P*-values less than 0.05 were regarded as statistically significant. Data analysis was performed using the Prism program (GraphPad Software, version 5.0d).

## Results

### GPR30 structure and subcellular localization

To monitor human GPR30, a receptor construct was made with the FLAG epitope at the receptor N-terminal end followed by a 6-amino acid linker, and the construct was transiently and stably expressed in HEK293 cells. An artificial signal sequence was inserted N-terminally of the FLAG epitope, which upon cleavage in the ER exposed the FLAG epitope immediately at the N-terminus (Figure 1A). The advantage of this construct is that it may be monitored by both mouse monoclonal M1 and M2 FLAG Ab, the former far superior for immunofluorescence staining, and the latter superior for immunoprecipitation and immunoblotting. A commercially available goat polyclonal GPR30 Ab made against the N-domain, and previously validated by us for receptor specificity by immunoblotting, immunoprecipitation, and immunofluorescence staining [15], was also used to monitor the receptor. Immunoprecipitation and immunoblotting of wild-type (WT) GPR30-transfected cells with M2 Ab revealed a complex pattern of receptor species with molecular masses of approximately 40 kDa, which is close to the theoretical mass of the receptor, 70, 110, and >150 kDa (Figure 1B).

Confocal immunofluorescence microscopy of fixed and permeabilized GPR30-expressing cells with M1 Ab showed that the total subcellular distribution of the receptor is also complex, with staining localized both in the PM and intracellularly in both a tubular-like network and distinct puncta, without any obvious nuclear staining (Figure 1C, *Dead*). A major portion of the staining overlapped with that of the ER marker calnexin (Figure 1D), a chaperone protein that retains unfolded and unassembled *N*-linked glycoproteins in the ER, and at least a portion of the receptor co-precipitated with this protein (Figure 1E). On the other hand, no co-staining occurred with the Golgi marker GM130 (Figure 1D). Thus, some of the intracellular GPR30 staining appears to represent unfolded and unassembled *N*-linked receptors in the ER.

Staining under live conditions showed that some GPR30 also reached the cell surface (Figure 1C, *Live*). Under these conditions, most of the staining appeared intracellularly as distinct puncta, indicating that the receptors that reach the cell surface undergo constitutive endocytosis, as previously reported [15,25]. To confirm constitutive endocytosis, live cells were also subjected to a reaction with the membrane-impermeable amine-reactive and thiol-cleavable biotinylation reagent sulpho-NHS-SS-biotin prior to receptor immunoprecipitation. As shown in Figure 1F (4°C), subjecting cells in this manner at 4°C prior to immunoprecipitation with M2 Ab revealed a specific biotinylated 70-kDa species in receptor-expressing cells but not in mock-transfected cells. Stripping the biotinylated cells with a reducing agent at 4°C prior to immunoprecipitation removed a majority of the biotin labeling of the 70-kDa receptor species, confirming cell surface localization of the species (Figure 1F, 4°C*+strip*). Incubating the biotinylated cells at 37°C for 30 min, to allow for any constitutive receptor endocytosis to occur, prior to stripping the cells at 4°C and immunoprecipitation increased the resistance of the 70-kDa species to stripping (Figure 1F, 37°C+*strip*). Together, these results show that the receptor reaches the cell surface as a 70-kDa species, which subsequently undergoes constitutive endocytosis.

**Figure 2:**
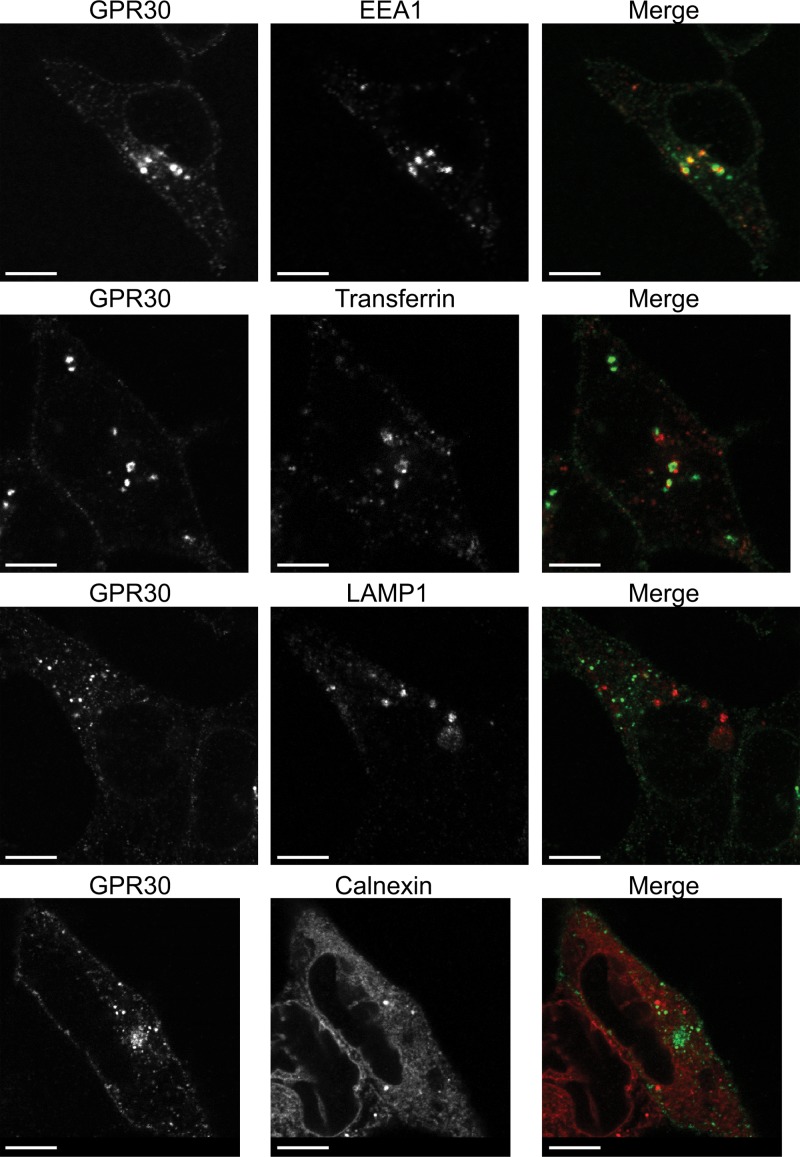
Subcellular trafficking of constitutively internalized GPR30 HEK cells transfected with GPR30 were incubated live with primary M1 FLAG Ab for 60 min at 37°C. The cells were then fixed and permeabilized prior to incubation with primary EEA1 Ab, LAMP1 Ab, or calnexin Ab. Secondary rabbit anti-mouse or mouse anti-rabbit ALEXA488- or ALEXA568-labeled Ab were then added. In the case of transferrin, Alexa568-labeled transferrin was added during the last 30 min of the live incubation with M1 FLAG Ab. The individual and merged (*Merge*) images were collected using a Nikon Eclipse confocal microscope, 60× objective. The results are representative of experiments performed at least three times. *Bar*, 10 μm.

Co-staining with various subcellular markers showed that the constitutively internalized receptors co-localized at least in part with the early endosomal marker EEA1 and partially with transferrin, a marker for recycling endosomes, whereas no co-staining was observed with the lysosomal marker LAMP1 or the ER marker calnexin (Figure 2). Thus, the intracellular pool of GPR30 is likely a combination of slowly maturing receptors in the ER and constitutively internalized receptors derived from the cell surface.

### Role of GPR30 *N*-glycosylation on receptor structure and activity

To determine if GPR30 is *N*-glycosylated, receptors immunoprecipitated with M2 Ab were treated with the *N*-glycosidase PNGase F. This treatment caused a complete loss of the 40-kDa species and an increase in the intensity of the 110-kDa species (Figure 1B). A partial decrease also occurred in the intensity of 70-kDa species, and two species appeared at approximately 30 and 37 kDa (Figure 1B). This result shows that GPR30 is *N*-glycosylated. Also, the change in reactivity of some of the species to Ab staining suggests a more complex structural impact of *N*-deglycosylation on the receptor N-domain.

**Figure 3:**
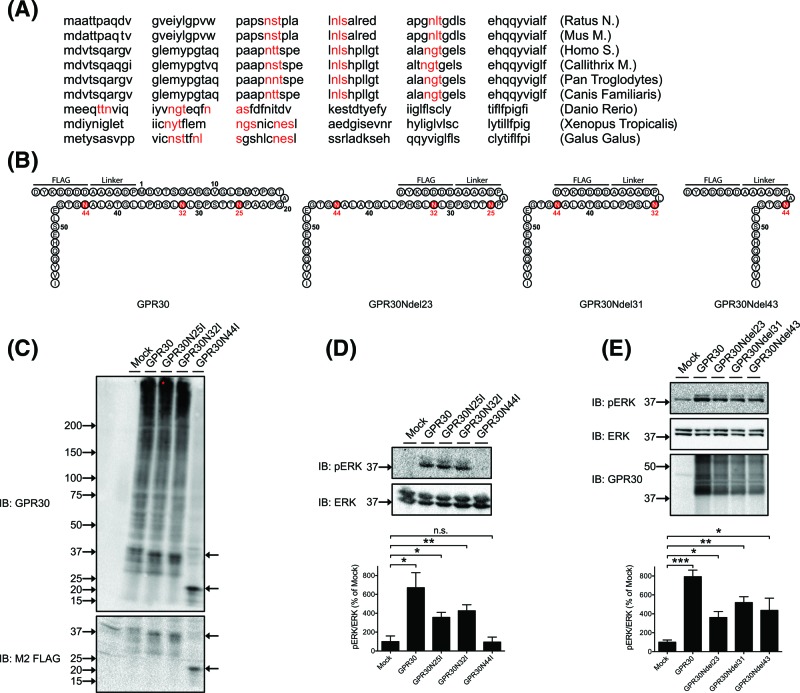
Structural and functional roles of GPR30 Asn^25^, Asn^32^, and Asn^44^ (**A**) Amino acid sequences of the GPR30 N-domain from different species, and with consensus *N*-glycosylation motifs indicated (*red*). (**B**) Schematic depiction of the GPR30 N-domain of WT and truncation mutant receptors used in the current study, and with asparagines mutated to isoleucine indicated (*red*). (**C**) HEK cells transfected without (*Mock*) or with GPR30, GPR30N25I, GPR30N32I, or GPR30N44I were lysed and IB with goat GPR30 Ab or M2 FLAG Ab. (**D**) Samples prepared in (C) were immunoblotted with pERK Ab and ERK Ab. (**E**) HEK cells transfected without (*Mock*) or with GPR30, GPR30Ndel23, GPR30Ndel31, or GPR30Ndel43 were immunoblotted with pERK Ab, ERK Ab, and GPR30 Ab. In (D,E), the pERK band intensities were normalized to the ERK band intensities, and the data are graphed as % of Mock. Molecular mass standards (*left side arrows*) and specific protein species (*right side arrows*) as discussed in ‘Results’ section are indicated, and the results are means ± S.E.M. of at least three independent experiments. *, *P*<0.05; **, *P*<0.01; ***, *P*<0.001, ns., not significant.

GPR30 contains three asparagines, Asn^25^, Asn^32^, and Asn^44^, in consensus sequences for *N*-glycosylation (Asn–X–Ser/Thr), all located in the extracellular N-domain, and these residues are highly conserved amongst mammals but less so between mammals and lower vertebrates (Figure 3A,B). To address the roles of these asparagines on receptor structure, activity, and maturation to the PM, each residue was mutated to isoleucine either individually to make GPR30N25I, GPR30N32I, and GPR30N44I, or in combination to make GPR30N25/32I. Direct immunoblotting of lysates from mock- and WT GPR30-transfected cells with the goat GPR30 Ab again revealed a complex receptor profile with the 40-kDa receptor species relatively clearly defined and a large range of higher molecular mass species (Figure 3C). The receptor peptide profiles of GPR30N25I and GPR30N32I (Figure 3C), as well as GPR30N25/N32I (data not shown), were essentially the same as that of WT GPR30. On the other hand, GPR30N44I was expressed as an apparently unique species at approximately 20 kDa, which was recognized by both the goat GPR30 Ab and M2 Ab (Figure 3C). GPR30N25I and GPR30N32I (Figure 3D), as well as GPR30N25/N32I (data not shown) also remained active, as determined by constitutive stimulation of ERK1/2 activity, whereas GPR30N44I was completely inactive (Figure 3D). To address the role of a larger portion of the GPR30 N-domain, without perturbing Asn^44^, GPR30 was truncated at Ala^23^ (GPR30Ndel23), Leu^31^ (GPR30Ndel31), and Ala^43^ (GPR30Ndel43) (Figure 3B). As shown in Figure 3E, the receptor peptide profiles of all the truncation mutants remained the same as that of WT GPR30, and all the mutants remained active. Together, these results show that N-domain residue Asn^44^ most likely is glycosylated, and that this modification is absolutely critical for receptor structure and activity. On the other hand, N-domain residues 1–42, including specifically residues Asn^25^ and Asn^32^, do not appear to play any major structural or functional role(s).

### Role of GPR30 *N*-glycosylation on receptor subcellular localization

**Figure 4:**
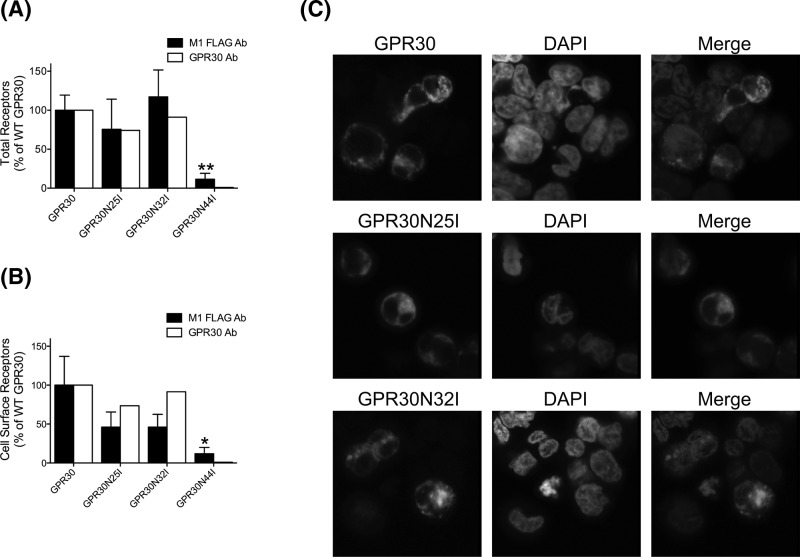
Roles of GPR30 Asn^25^ and Asn^32^ in receptor subcellular distribution HEK cells transfected with GPR30, GPR30N25I, GPR30N32I, or GPR30N44I were stained following permeabilization (*Total Receptors*) (**A**) or live (*Cell Surface Receptors*) (**B**) with M1 FLAG Ab (*filled bars*) or GPR30 Ab (*open bars*), and then subjected to flow cytometry. The data are graphed as % of WT GPR30. (**C**) HEK cells transfected with GPR30, GPR30N25I, or GPR30N32I were fixed and permeabilized prior to incubating with M1 FLAG Ab and then with rabbit anti-mouse ALEXA488-labeled Ab. DAPI was used for nuclear staining. The individual and merged (*Merge*) images were collected using a Nikon Eclipse confocal microscope, 60× objective. In (A,B), the results are either the means ± S.E.M. of at least three independent experiments (*M1 FLAG Ab*) or the means of two independent experiments (*GPR30 Ab*). *, *P*<0.05; **, *P*<0.01.

To investigate the roles of Asn^25^, Asn^32^, and Asn^44^ on receptor subcellular localization, cells expressing WT GPR30, GPR30N25I, GPR30N32I, and GPR30N44I were first monitored by flow cytometry with M1 Ab. GPR30N25I and GPR30N32I were recognized by this Ab on both permeabilized and non-permeabilized cells, revealing total (Figure 4A) and cell surface expressions (Figure 4B) not significantly different from that of WT GPR30. On the other hand, GPR30N44I was not recognized by M1 Ab either on permeabilized or non-permeabilized cells. This is in contrast with immunoblotting, where GPR30N44I was recognized by both M2 Ab and the goat GPR30 Ab (Figure 3C). As M2 Ab is not recommended for immunofluorescence staining because of non-specific effects, we instead performed flow cytometry with the goat GPR30 Ab. GPR30N25I and GPR30N32I were also recognized by this antibody, again revealing a very similar total (Figure 4A) and cell surface expression (Figure 4B) to WT GPR30. On the other hand, this Ab did not recognize GPR30N44I either.

We also investigated the total subcellular distribution of the receptor mutants by confocal microscopy with M1 Ab in fixed and permeabilized cells. Again, the total distributions of GPR30N25I and GPR30N32I were very similar to that of WT GPR30 with immunoreactivity apparently on both plasma and intracellular membranes (Figure 4C), whereas neither M1 Ab nor the goat GPR30 Ab recognized GPR30N44I (data not shown). We conclude from these results that Asn^25^ and Asn^32^ are not required for the maturation of the receptor. On the other hand, the role of Asn^44^ in receptor maturation could not be determined as mutation of this residue produces a receptor product that is only recognized by N-domain Ab in a denatured state.

## Discussion

The subcellular localization of GPR30 activity has long been debated, but only very few studies have been done to directly address this issue. Here, we used three antibodies specifically targetting the receptor N-domain and various N-domain mutants to investigate the role of receptor *N*-glycosylation. Treatment with PNGase F showed that the receptor is *N*-glycosylated. Following mutation of Asn^44^, the receptor was recognized by Ab only in a denatured state as a 20-kDa species, and this species was inactive as determined by constitutive stimulation of ERK1/2 signaling. On the other hand, mutation of Asn^25^ and Asn^32^ either individually or in combination, or successive truncation of N-domain residues 1–42, had no apparent effect on either receptor structure, activity, or maturation. We conclude from these results that Asn^44^ is *N*-glycosylated and critical for the maturation of an active receptor to the PM, whereas N-domain residues 1–42 do not have any major structural or functional roles.

Direct immunoblotting of cell lysates and immunofluorescence microscopy of fixed cells showed that the expression and subcellular localization of the receptor are complex with receptor species ranging from approximately 40 kDa to higher masses and localized in the ER, PM, and endocytic vesicles. Immunoprecipitation followed by immunoblotting more clearly identified some major species at approximately 40, 70, and 110 kDa. Fluorescence microscopy, flow cytometry, and cell surface biotinylation revealed that the receptor appears to reach the PM as a 70-kDa species, which subsequently undergoes constitutive endocytosis.

The theoretical mass of GPR30 is approximately 40 kDa, and higher mass species could be glycosylated or detergent-resistant oligomeric receptor forms, whereas lower mass species could be proteolytically degraded forms. Protease inhibitors were included in all sample preparations, arguing that any proteolysis must have occurred primarily intracellularly before preparation. Receptor oligomers have not been addressed, but the receptor forms PDZ-dependent complexes with the scaffold proteins SAP97 and PSD-95 [7,8,16,17], NHERF-1 [26], and an apparently PDZ-independent complex with receptor-activity modifying protein (RAMP) 3 [27]. However, the detergent-resistant nature of these complexes is not known.

PNGase F treatment showed that the receptor is *N*-glycosylated. Many class A GPCRs migrate on SDS/PAGE at 50–70 kDa in their glycosylated state and at 30–40 kDa following deglycosylation, the decrease primarily due to changed SDS binding to the protein rather than an absolute loss of protein mass. Considering that the receptor resides on the cell surface as a 70-kDa species, typical of GPCR, and that GPCRs are normally *N*-glycosylated in this location, the partial decrease in immunoreactivity of this species, and the appearance of the two species at approximately 30 and 37 kDa following PNGase F treatment, argues that the 70-kDa species is *N*-glycosylated. The change in reactivity of the 40-kDa species could indicate that it is also *N*-glycosylated. However, the complete loss in reactivity of this species, and the increase in reactivity of the 110-kDa species, suggest a different impact of *N*-deglycosylation, possibly involving N-domain availability.

Asn^25^, Asn^32^, and Asn^44^ in the N-domain are the only residues that can be *N*-glycosylated in GPR30. Successive truncation of receptor N-domain residues 1–42, or specific mutation of Asn^25^ and Asn^32^, had no major effects on either the structural profile, maturation, or activity of the receptor, suggesting that these residues more likely do not play major structural or functional roles. On the other hand, the decrease in both relative mass and activity of the receptor following mutation of Asn^44^ argue that this residue is *N*-glycosylated, and that this modification is necessary for the receptor to form higher mass complexes. Whether the decrease in receptor mass upon mutation of this residue is simply due to *N*-deglycosylation or also to intracellular degradation is unclear. Lack of *N*-glycosylation could perturb receptor folding in the ER, possibly leading to ER-associated degradation. Interestingly, Asn^44^ mutation yielded a receptor product that was not recognized by Ab in a non-denatured form, which unfortunately prevented us from determining the subcellular localization of the product by flow cytometry and immunofluorescence microscopy. On the other hand, the product was still recognized by the same Ab in a denatured form by immunoblotting, indicating that the N-domain of the product is intact. Considering this, one may speculate that under non-denaturing conditions the loss of N-domain immunoreactivity following *N*-deglycosylation by Asn^44^ mutation and the rather unique changes in N-domain immunoreactivity of some receptor species following *N*-deglycosylation by PNGase F treatment are related, involving a change in N-domain orientation and availability.

Only one other study has addressed the role of *N*-glycosylation in GPR30 [18]. In this study, the investigators reported that blocking receptor *N*-glycosylation with tunicamycin resulted in nuclear localization of the receptor as determined by fluorescence imaging of M2 Ab-stained cells. Mutating both Asn^25^ and Asn^32^ yielded the same subcellular distribution. Curiously, these investigators did not study the role of Asn^44^. Nevertheless, they concluded from their results that *N*-glycosylation of GPR30 is a regulated event controlling the nuclear localization and function of the receptor as a transcription factor. While these observations are interesting, we found no evidence that mutation of Asn^25^ and Asn^32^ either individually or in combination caused either nuclear localization or altered activity of the receptor.

In summary, we conclude from our results that GPR30 N-domain residue Asn^44^ is *N*-glycosylated and absolutely critical for the maturation of an active receptor to the PM, whereas N-domain residues 1–42 are dispensable in this regard. These results support that GPR30 is active in the PM, shedding further light on the debate about the subcellular localization of receptor activity.

## Abbreviations

DMEM: Dulbecco’s modified Eagle’s medium
ER: endoplasmatic reticulum
ERK: extracellular-regulated protein kinase
Gi/o: G protein Gi/o
GPCR: G protein-coupled receptor
GPR30: GPCR 30
Gs: G protein Gs
HEK: human embryonic kidney
LAMP1: Lysosomal-associated membrane protein 1
MAGUK: membrane-associated guanylate kinase
PDZ: PSD-95/Discs-large/ZO-1 homology
PNGase F: Peptide:N-glycosidase F
PM: plasma membrane
PSD-95: postsynaptic density-95
SAP97: synapse-associated protein 97
WT: wild-type
